# Acute Kidney Injury Network: report of an initiative to improve outcomes in acute kidney injury

**DOI:** 10.1186/cc5713

**Published:** 2007-03-01

**Authors:** Ravindra L Mehta, John A Kellum, Sudhir V Shah, Bruce A Molitoris, Claudio Ronco, David G Warnock, Adeera Levin, the Acute Kidney Injury Network

**Affiliations:** 1 Department of Medicine, University of California San Diego Medical Center 8342, 200 W. Arbor Drive, San Diego, CA 92103, USA; 2 Department of Critical Care Medicine, University of Pittsburgh, 3550 Terrace Street, 608 Scaife Hall, Pittsburgh, PA 15261, USA; 3 Division of Nephrology, UAMS College of Medicine, 4301 West Markham, Slot 501, Little Rock, AR 72205, USA; 4 Department of Medicine, Indiana University, Indianapolis, IN, USA; 5 Department of Nephrology Dialysis & Transplantation, San Bortolo Hospital, Vicenza, Italy; 6 Department of Medicine, University of Alabama, 1900 University Blvd, Birmingham, AL, USA; 7 Department of Medicine, University of British Columbia, St Pauls Hospital, 1160 Burrard St, Vancouver BC, V6Z1Y8, Canada

## Abstract

**Introduction:**

Acute kidney injury (AKI) is a complex disorder for which currently there is no accepted definition. Having a uniform standard for diagnosing and classifying AKI would enhance our ability to manage these patients. Future clinical and translational research in AKI will require collaborative networks of investigators drawn from various disciplines, dissemination of information via multidisciplinary joint conferences and publications, and improved translation of knowledge from pre-clinical research. We describe an initiative to develop uniform standards for defining and classifying AKI and to establish a forum for multidisciplinary interaction to improve care for patients with or at risk for AKI.

**Methods:**

Members representing key societies in critical care and nephrology along with additional experts in adult and pediatric AKI participated in a two day conference in Amsterdam, The Netherlands, in September 2005 and were assigned to one of three workgroups. Each group's discussions formed the basis for draft recommendations that were later refined and improved during discussion with the larger group. Dissenting opinions were also noted. The final draft recommendations were circulated to all participants and subsequently agreed upon as the consensus recommendations for this report. Participating societies endorsed the recommendations and agreed to help disseminate the results.

**Results:**

The term AKI is proposed to represent the entire spectrum of acute renal failure. Diagnostic criteria for AKI are proposed based on acute alterations in serum creatinine or urine output. A staging system for AKI which reflects quantitative changes in serum creatinine and urine output has been developed.

**Conclusion:**

We describe the formation of a multidisciplinary collaborative network focused on AKI. We have proposed uniform standards for diagnosing and classifying AKI which will need to be validated in future studies. The Acute Kidney Injury Network offers a mechanism for proceeding with efforts to improve patient outcomes.

## Introduction

Acute renal failure (ARF) is a complex disorder that occurs in a variety of settings with clinical manifestations ranging from a minimal elevation in serum creatinine to anuric renal failure. It is often under-recognized and is associated with severe consequences [1-4]. Recent epidemiological studies demonstrate the wide variation in etiologies and risk factors [1,5-7], describe the increased mortality associated with this disease (particularly when dialysis is required) [1,4,6,8,9], and suggest a relationship to the subsequent development of chronic kidney disease (CKD) and progression to dialysis dependency [1,4,8,10-12]. Emerging evidence suggests that even minor changes in serum creatinine are associated with increased in-patient mortality [13-20]. ARF has been the focus of extensive clinical and basic research efforts over the last decades. The lack of a universally recognized definition of ARF has posed a significant limitation. Despite the significant progress made in understanding the biology and mechanism of ARF in animal models, translation of this knowledge into improved management and outcomes for patients has been limited.

During the last five years, several groups have recognized these limitations and have worked to identify the knowledge gaps and define the necessary steps to correct these deficiencies. These efforts have included consensus conferences and publications from the Acute Dialysis Quality Initiative (ADQI) group [19,21-25], the American Society of Nephrology (ASN) ARF Advisory group [26], the International Society of Nephrology (ISN), and the National Kidney Foundation (NKF) and KDIGO (Kidney Disease: Improving Global Outcomes) groups [27]. Additionally, the critical care societies have developed formal intersociety collaborations such as the International Consensus Conferences in Critical Care [28]. Recognizing that future clinical and translational research in ARF will require multidisciplinary collaborative networks, the ADQI group and representatives from three nephrology societies (ASN, ISN, and NKF) and the European Society of Intensive Care Medicine met in Vicenza, Italy, in September 2004. They proposed the term acute kidney injury (AKI) to reflect the entire spectrum of ARF, recognizing that an acute decline in kidney function is often secondary to an injury that causes functional or structural changes in the kidneys. The group established the Acute Kidney Injury Network (AKIN) as an independent collaborative network comprised of experts selected by the participating societies to represent both their area of expertise and their sponsoring organization. AKIN is intended to facilitate international, interdisciplinary, and intersocietal collaborations to ensure progress in the field of AKI and obtain the best outcomes for patients with or at risk for AKI.

This report describes an interim definition and staging system for AKI and a plan for further activities of the collaborative network which were developed at the first AKIN conference held in Amsterdam, The Netherlands, in September 2005.

## Materials and methods

Representatives of the major critical care and nephrology societies and associations and invited content experts were assigned to workgroups to consider three topics: (a) the development of uniform standards for definition and classification of AKI, (b) joint conference topics, and (c) the interdisciplinary collaborative research network. Each workgroup had an assigned chair and co-chair to facilitate the discussion and develop summary recommendations of the workgroup. The draft recommendations were then refined and improved during discussion with the larger group. Key points and issues were noted and then discussed a second time if no resolution was reached initially. When a majority view was not evident or when the area was felt to be of extreme importance, votes were tallied. Dissenting opinions were also noted. The final recommendations were circulated to all participants and subsequently agreed upon as the consensus recommendations for this report. After an iterative process of revisions, the final manuscript was presented to each of the respective societies for endorsement. Societies were asked to facilitate dissemination of the findings to their membership through presentations in society conferences and publication of summary reports in society journals, Web sites, and other forms of communication.

## Results

### 1. Proposal for uniform standards for definition and classification of AKI

#### Definition and diagnostic criteria of AKI

For any condition, the clinician needs to know whether the disease is present and, if so, where and when the patient falls in the natural history of the disease. The former facilitates recognition whereas the latter defines time points for intervention. Unfortunately, there has been no uniformly accepted definition of AKI. Studies describe ARF or AKI based on serum creatinine changes, absolute levels of serum creatinine, changes in blood urea nitrogen or urine output, or the need for dialysis [1,11,20,29-36]. The wide variation in definitions has made it difficult to compare information across studies and populations [37].

#### Diagnostic criteria

Recognition of AKI requires the delineation of easily measured criteria that can be widely applied. Serum creatinine levels and changes in urine output are the most commonly applied measures of renal function; however, they are each influenced by factors other than the glomerular filtration rate (GFR) and do not provide any information about the nature or site of kidney injury. The proposed diagnostic criteria (Table 1) were based on consideration of the following concepts:

**Table 1:** Diagnostic criteria for acute kidney injury

An abrupt (within 48 hours) reduction in kidney function currently defined as an absolute increase in serum creatinine of more than or equal to 0.3 mg/dl (≥ 26.4 μmol/l), a percentage increase in serum creatinine of more than or equal to 50% (1.5-fold from baseline), or a reduction in urine output (documented oliguria of less than 0.5 ml/kg per hour for more than six hours).

The above criteria include both an absolute and a percentage change in creatinine to accommodate variations related to age, gender, and body mass index and to reduce the need for a baseline creatinine but do require at least two creatinine values within 48 hours. The urine output criterion was included based on the predictive importance of this measure but with the awareness that urine outputs may not be measured routinely in non-intensive care unit settings. It is assumed that the diagnosis based on the urine output criterion alone will require exclusion of urinary tract obstructions that reduce urine output or of other easily reversible causes of reduced urine output. The above criteria should be used in the context of the clinical presentation and following adequate fluid resuscitation when applicable. Note: Many acute kidney diseases exist, and some (but not all) of them may result in acute kidney injury (AKI). Because diagnostic criteria are not documented, some cases of AKI may not be diagnosed. Furthermore, AKI may be superimposed on or lead to chronic kidney disease.

1. The definition needs to be broad enough to accommodate variations in clinical presentation over age groups, locations, and clinical situations.

2. Sensitive and specific markers for kidney injury are not currently available in clinical practice. Several groups are working on developing and validating biomarkers of kidney injury and GFR which may be used in the future for diagnosis and prognosis.

3. There is accumulating evidence that small increments in serum creatinine are associated, in a variety of settings, with adverse outcomes [13-20] that are manifest in short-term morbidity and mortality and in longer-term outcomes, including 1-year mortality [15-17]. Current clinical practice does not focus much attention on small increments in serum creatinine, which are often attributed to lab variations. However, the coefficient of variation of serum creatinine with modern analyzers is relatively small and therefore increments of 0.3 mg/dl (25 μmol/l) are unlikely to be due to assay variation [38]. Changes in volume status can influence serum creatinine levels [39]. Because the amount of fluid resuscitation depends on the underlying clinical situation [40], the group agreed that application of the diagnostic criteria would be used only after an optimal state of hydration had been achieved.

4. A time constraint of 48 hours for diagnosis was selected based on the evidence that adverse outcomes with small changes in creatinine were observed when the creatinine elevation occurred within 24 to 48 hours [15,16] and to ensure that the process was acute and representative of events within a clinically relevant time period. In the two aforementioned studies, there was no distinction of underlying CKD or *de novo *AKI. However, in the study by Chertow and colleagues [13], the odds ratio for mortality with a change in creatinine of 0.3 mg/dl (25 μmol/l) was 4.1 (confidence interval 3.1 to 5.5) adjusting for CKD. There is no requirement to wait 48 hours to diagnose AKI or initiate appropriate measures to treat AKI. Instead, the time period is designed to eliminate situations in which the increase in serum creatinine by 0.3 is very slow and thus is not 'acute.'

5. It was recognized that AKI is often superimposed on pre-existing CKD. Further validation will be required to determine whether the criterion of a creatinine elevation of 0.3 mg/dl (25 μmol/l) is applicable to these patients (that is, whether a creatinine increase of more than 0.3 mg/dl from an elevated baseline represents AKI and has the same risks as a creatinine increase from a normal baseline).

6. The need for including urine output as a diagnostic criterion is based on the knowledge of critically ill patients in whom this parameter often heralds renal dysfunction before serum creatinine increases.

A minority of group members, both intensivists and nephrologists, felt that a urine output reduction of less than 0.5 ml/kg per hour over the span of six hours was not specific enough to lead confidently to the designation of AKI. It was recognized that the hydration state, use of diuretics, and presence of obstruction could influence the urine volume, hence the need to consider the clinical context. Additionally, accurate measurements of urine output may not be easily available in all cases, particularly in patients in non-intensive care unit settings. Despite these limitations, it was felt that the use of changes in urine offers a sensitive and easily discernible means of identifying patients, but its value as an independent criterion for diagnosis of AKI will need to be validated.

The proposed diagnostic criteria for AKI are designed to facilitate acquisition of new knowledge and validate the emerging concept that small alterations in kidney function may contribute to adverse outcomes. The goal of adopting these explicit diagnostic criteria is to increase the clinical awareness and diagnosis of AKI. It is recognized that there may be an increase in false-positives, so that some patients labeled with AKI will not have the condition. There was consensus that adopting the more inclusive criteria is preferable to the current situation, in which the condition is under-recognized and many people are identified late in the course of their illness and potentially miss the opportunity for prevention or application of strategies to minimize further kidney damage.

#### Staging/classification

The goal of a staging system is to classify the course of a disease in a reproducible manner that supports accurate identification and prognostication and informs diagnostic or therapeutic interventions. The group recognized that a number of systems for staging and classifying AKI are currently in use or have been proposed [41]. The RIFLE (Risk, Injury, Failure, Loss, and End-stage kidney disease) criteria [25] proposed by the ADQI group were developed by an interdisciplinary, international consensus process and are now being validated by different groups worldwide [36,37]. However, according to data that have emerged since then, smaller changes in serum creatinine than those considered in the RIFLE criteria might be associated with adverse outcomes [13-18]. Additionally, given the consensus definition for AKI (Table 1), RIFLE criteria have been modified so that patients meeting the definition for AKI could be staged (Table 2). The proposed staging system retains the emphasis on changes in serum creatinine and urine output but includes the following principles:

**Table 2:** Classification/staging system for acute kidney injury^a^

Stage	Serum creatinine criteria	Urine output criteria
1	Increase in serum creatinine of more than or equal to 0.3 mg/dl (≥ 26.4 μmol/l) or increase to more than or equal to 150% to 200% (1.5- to 2-fold) from baseline	Less than 0.5 ml/kg per hour for more than 6 hours
2^b^	Increase in serum creatinine to more than 200% to 300% (> 2- to 3-fold) from baseline	Less than 0.5 ml/kg per hour for more than 12 hours
3^c^	Increase in serum creatinine to more than 300% (> 3-fold) from baseline (or serum creatinine of more than or equal to 4.0 mg/dl [≥ 354 μmol/l] with an acute increase of at least 0.5 mg/dl [44 μmol/l])	Less than 0.3 ml/kg per hour for 24 hours or anuria for 12 hours

^a^Modified from RIFLE (Risk, Injury, Failure, Loss, and End-stage kidney disease) criteria [26]. The staging system proposed is a highly sensitive interim staging system and is based on recent data indicating that a small change in serum creatinine influences outcome. Only one criterion (creatinine or urine output) has to be fulfilled to qualify for a stage. ^b^200% to 300% increase = 2- to 3-fold increase. ^c^Given wide variation in indications and timing of initiation of renal replacement therapy (RRT), individuals who receive RRT are considered to have met the criteria for stage 3 irrespective of the stage they are in at the time of RRT.

1. Although diagnosis of AKI is based on changes over the course of 48 hours, staging occurs over a slightly longer time frame. One week was proposed by the ADQI group in the original RIFLE criteria [25].

2. There was a conscious decision not to include the therapy for AKI (that is, renal replacement therapy [RRT]) as a distinct stage because this constitutes an outcome of AKI.

3. The new staging system maps to the RIFLE stages as follows:

3a. RIFLE 'Risk' category should have the same criteria as for the diagnosis of stage 1 AKI.

3b. Those who are classified as having 'Injury' and 'Failure' categories map to stages 2 and 3 of AKI.

3c. The 'Loss' and 'End-stage kidney disease' categories were removed from the staging system and remain outcomes.

3d. Given the variability inherent in commencing RRT and due to variability in resources in different populations and countries, patients receiving RRT are to be included in stage 3 (analogous to stage 5 CKD, GFR of less than 15, or dialysis).

### 2. Future joint conference topics and key collaborative research questions

There is a need to ensure that collaborative and integrated joint conferences are planned to facilitate the dissemination of knowledge, clarify clinical practice, and enhance research. Many organizations are currently in the process of planning meetings on ARF/AKI. These meeting take various forms: knowledge exchange/scientific meetings, consensus controversies, and research initiatives. The group described five key topics that should be addressed by any of the professional communities involved in the care of patients with AKI. The particular venue and the process and products of these conferences were not discussed in detail. An overview of the topics and issues that would be well served by a multidisciplinary consensus or controversies conference is presented in Table 3. These topics reflect important areas in which there is a need for ongoing research to develop evidence. A key step for future conferences will be to determine which research questions are most important and pressing to advance the field and improve outcomes from AKI.

**Table 3:** Potential topics identified for future consensus conferences

	Subject	Topics
1.	Epidemiology of AKI	What is a 'nomenclature' that is based on simple, universally available data and that can identify all patients globally with AKI irrespective of location and age? What are the data to help determine etiology once AKI is identified? What are the correlates of AKI in regard to pathology/physiology? Is there a validated method for assessing severity of AKI separate from multiple organ failure? What is the relationship between degree of severity and outcomes?
2.	Outcomes from AKI	What are the clinically meaningful outcomes that are important in clinical studies of AKI?
3.	Strategies to change outcomes	Prevention Treatment
		Non-dialytic
		Dialysis
		Timing of initiation
		Modality selection (CRRT, IHD, PD)
		Intensity of therapy (dose)
		Cessation of renal replacement therapy
4.	Data needed to advance knowledge in AKI	Datasets collected at contact with health care system Intensive care unit admission Biological sample repositories
5	Process outcomes	Measures of effectiveness of current processes for changing behavior/attitude of caregivers and ultimately patient outcomes from AKI.

AKI, acute kidney injury; CRRT, continuous renal replacement therapy; IHD, intermittent hemodialysis; PD, peritoneal dialysis.

### 3. Need for an international collaborative network

AKI is a global problem with varying etiologies and manifestations, but the outcomes are similar [1-4,6]. Given the wide global variation in the natural history and management of AKI, it is essential that mechanisms for sharing information and for collaboration among centers be developed. It was felt that the establishment of an international collaborative research effort for AKI would contribute to international research and education about AKI. The group proposed four major topics that would need to be addressed by this initiative (Table 4).

**Table 4:** Recommendations for establishing a collaborative network for acute kidney injury (AKI) research

	Component	Principles and approach
1.	Identify the key roles of the participating groups	a. The collaborative effort should be inclusive and open to all interested societies/organizations. b. Participation in the collaborative organization will require commitment of time, expertise, and/or resources as appropriate to the specific initiative and in accordance with the means of the organization/group. c. An organizational structure will be required to coordinate the activities. d. Work products from the collaborative effort will require a mechanism for recognizing the contributions of each group.
2.	Scope of collaborations	a. Identify topics in AKI areas of mutual interest and of wide application. b. Develop consensus statements for best practice where there is limited or no evidence and where, due to accepted practices, it will be difficult to get evidence. c. Develop tools to standardize the management of AKI. d. Develop evidence through clinical research where feasible. e. Develop practice recommendations/guidelines. f. Implement guidelines.
3.	Define infrastructure needs	a. Identify key components needed (for example, database, protocols for Web-based information transfer). b. Establish the requirements for sharing information with regulatory agencies. c. Define training needs for developing researchers and the resources that are required and define what hurdles will need to be overcome. d. International collaboration will require identification of peer-reviewed, public, and commercial sources of financial support. e. Develop an inventory of current collaborative efforts and establish relationships with these existing networks.
4.	Identify common unifying principles that would form the basis of ongoing collaboration	a. Establish protocols for consistent data entry that allows benchmarking of participating units. b. Identify questions that interest the majority of the participants. c. Initiate a short-term collaborative project to validate proposed AKI definition as an initial project.

## Conclusion

AKI is a complex disorder for which there is no currently accepted uniform definition. Having a standard for diagnosing and classifying AKI would enhance our ability to improve the management of these patients. We have described the formation of a multidisciplinary collaborative network focused on AKI and have proposed uniform standards for diagnosing and classifying AKI. The proposed standards will need to be validated in future studies. These standards build upon existing knowledge and permit individuals using current staging systems (for example, RIFLE) to transition to the new system without loss of comparability. These recommendations have been endorsed by the participating societies, which represent the majority of the critical care and nephrology societies worldwide and which have been asked to disseminate the results via presentations at the national and regional society conferences and through publication of summary reports in society journals (see Table 5 for society endorsement details). We believe that these recommendations provide a stepping stone to standardizing the care of patients with AKI and will greatly enhance our ability to design prospective studies to evaluate potential prevention and treatment strategies. One of the limitations of consensus recommendations is that they are often not adopted. We anticipate that the broad support and commitment obtained through society involvement will significantly enhance the ability to disseminate the results to the worldwide community and to address this limitation. Future clinical and translational research in AKI will require the development of collaborative networks of investigators drawn from various disciplines to facilitate the acquisition of evidence through well-designed and well-conducted clinical trials, dissemination of information via multidisciplinary joint conferences and publications, and improvement of the translation of knowledge from pre-clinical research. We anticipate that the AKIN will provide an effective mechanism for facilitating efforts to improve patient outcomes.

**Table 5:** Acute Kidney Injury Network summit meeting participants and workgroups

Name	Representation	Joint conference	Interdisciplinary collaborative research network	Interim proposals for terminology, diagnosis, classification, and staging
Miet Schetz	Acute Dialysis Quality Initiative		X	
Sudhir V Shah	ASN	X (co-chair)		
Bruce A Molitoris	ASN		X	
Aysin Bakkaloglu	IPNA	X		
Arvind Bagga	IPNA		X	
Prasad Devarajan	American Society of Pediatric Nephrologists			X
Raul Lombardi	SLANH		X	
Emmanuel A Burdmann	SLANH	X		
Kai-Uwe Eckardt	European Dialysis and Transplant Association-European Renal Association		X (co-chair)	
Claudio Ronco	International Society of Nephrology	X		
Ravindra L Mehta	International Society of Nephrology			X (co-chair)
Adeera Levin	NKF	X		
David G Warnock	NKF	X		
Ashok Kirpalani	Indian Society of Nephrology			X
Haiyan Wang	CSN			X
Yipu Chen	CSN	X		
Vince D'Intini	Asian Pacific Society of Nephrology		X	
Michael Joannidis	European Society of Intensive Care Medicine		X	
Charles G Durbin Jr.	Society of Critical Care Medicine		X (co-chair)	
Patrick SK Tan	Asia Pacific Association of Critical Care Medicine		X	
Constantine Manthous	American Thoracic Society	X (co-chair)		
Claude Guerin	French Society			X
Frederique Schortgen	French Society		X	
John A Kellum	American College of Chest Physicians			X (co-chair)
Steve Webb	ANZICS	X		
Geoff Dobb	ANZICS		X	
Jean-Roger Le Gall	Expert			X
Eric Hoste	Expert		X	
Andrea Lassnigg	Expert			X
William Macias	Expert			X
Stefan Herget-Rosenthal	Expert			X
Joseph V Bonventre	Expert			X

ANZICS, Australian and New Zealand Intensive Care Society; ASN, American Society of Nephrology; CSN, Chinese Society of Nephrology; IPNA, International Pediatric Nephrology Association; NKF, National Kidney Foundation; SLANH, Sociedade Latino-Americana de Nefrologia e Hipertensão.

## Key messages

• AKI is a complex disorder, and we have proposed uniform standards for diagnosing and classifying AKI on the basis of existing systems (that is, RIFLE). These proposals will require validation.

• Our recommendations have been endorsed by participating societies that represent the majority of critical care and nephrology societies worldwide.

• These recommendations provide a stepping stone to standardizing the care of patients with AKI and will greatly enhance our ability to design prospective studies to evaluate potential prevention and treatment strategies.

• Future clinical and translational research in AKI will require the development of collaborative networks. The AKIN was formed to provide an effective mechanism for facilitating such efforts.

## Abbreviations

ADQI = Acute Dialysis Quality Initiative; AKI = acute kidney injury; AKIN = Acute Kidney Injury Network; ARF = acute renal failure; ASN = American Society of Nephrology; CKD = chronic kidney disease; GFR = glomerular filtration rate; ISN = International Society of Nephrology; NKF = National Kidney Foundation; RIFLE = Risk, Injury, Failure, Loss, and End-stage kidney disease; RRT = renal replacement therapy.

## Competing interests

The authors declare that they have no competing interests.

## Authors' contributions

All authors participated in the AKIN conference workgroups, development of the summary statement, and review of the manuscript. All authors read and approved the final manuscript.
